# Feasting, not fasting: winter diets of cave hibernating bats in the United States

**DOI:** 10.1186/s12983-021-00434-9

**Published:** 2021-09-23

**Authors:** Riley F. Bernard, Emma V. Willcox, Reilly T. Jackson, Veronica A. Brown, Gary F. McCracken

**Affiliations:** 1 Department of Zoology and Physiology, University of Wyoming, Laramie, 82071 USA; 2 Department of Forestry, Wildlife and Fisheries, University of Tennessee, Knoxville, 37996 USA; 3 Department of Ecology and Evolutionary Biology, University of Tennessee, Knoxville, 37996 USA; 4 Department of Biological Sciences, University of Arkansas, Fayetteville, 72701 USA; 5 Center for Environmental Biotechnology, University of Tennessee, Knoxville, 37996 USA

**Keywords:** Chiroptera, Foraging, Ion Torrent, Hibernation, Insects, MiSeq, Winter diet

## Abstract

Temperate bat species use extended torpor to conserve energy when ambient temperatures are low and food resources are scarce. Previous research suggests that migratory bat species and species known to roost in thermally unstable locations, such as those that roost in trees, are more likely to remain active during winter. However, hibernating colonies of cave roosting bats in the southeastern United States may also be active and emerge from caves throughout the hibernation period. We report what bats are eating during these bouts of winter activity. We captured 2,044 bats of 10 species that emerged from six hibernacula over the course of 5 winters (October–April 2012/2013, 2013/2014, 2015/2016, 2016/2017, and 2017/2018). Using Next Generation sequencing of DNA from 284 fecal samples, we determined bats consumed at least 14 Orders of insect prey while active. Dietary composition did not vary among bat species; however, we did record variation in the dominant prey items represented in species’ diets. We recorded Lepidoptera in the diet of 72.2% of individual *Corynorhinus rafinesquii* and 67.4% of individual *Lasiurus borealis*. Diptera were recorded in 32.4% of *Myotis leibii*, 37.4% of *M. lucifugus*, 35.5% of *M. sodalis* and 68.8% of *Perimyotis subflavus*. Our study is the first to use molecular genetic techniques to identify the winter diet of North American hibernating bats. The information from this study is integral to managing the landscape around bat hibernacula for insect prey, particularly in areas where hibernating bat populations are threatened by white-nose syndrome.

**Supplementary Information:**

The online version contains supplementary material available at 10.1186/s12983-021-00434-9.

## Introduction

The low ambient temperatures experienced by temperate insectivorous bats during winter pose two energetic challenges. First, due to their small size and associated high surface area-to-volume ratio, cold winter conditions make bats susceptible to high levels of heat loss and energy expenditure [1–3]. Second, once expended, their energy reserves are not easily replenished, as prey availability in winter is limited as low ambient temperature reduces prey activity and abundance [1, 4–6]. Therefore, temperate bats reduce their energetic costs in winter by engaging in periodic bouts of torpor, a physiological state characterized by low body temperature and metabolic rate, for prolonged periods of time [1, 7, 8].

Due to the physiological constraints of hibernation, hibernating mammals, including temperate bats [9], must periodically arouse to maintain homeostasis, with typical activities being urination and drinking of water [10, 11]. At higher temperate latitudes, where daily temperatures during winter rarely rise above freezing and there is little opportunity to feed due to scarcity of prey [7, 12, 13], bats must survive on energy stored as fat and protein [2]. Under these conditions, arousals occur but are relatively infrequent [14–16]. In contrast, at lower latitudes where winters are milder, the hibernation season is shorter and prey abundance and activity are greater [17]. Therefore, winter foraging may provide bats the opportunity to replenish energy stores [2]. Periodic arousals and activity outside hibernacula during winter provide evidence for winter foraging both in North American and European bat species [7, 13, 18–24].

Although optimal hibernation theory [2] suggests that sporadic foraging can be advantageous for bats hibernating in milder temperate climates, it may become particularly important for North American cave-hibernating species suffering from the disease white-nose syndrome (WNS). *Pseudogymnoascus destructans* (*Pd*)*,* the fungal agent that causes WNS, invades the cutaneous membrane of a bat’s muzzle, wings, ears and tail membrane during hibernation, eroding the epidermis and underlying skin and connective tissue [25, 26]. Once invasion occurs, the fungus disrupts torpor cycles and metabolic processes leading to the depletion of energy reserves through increased arousals, ultimately leading to increased morbidity and mortality during winter [27–29]. *Pseudogymnoascus destructans* was first detected in the southeastern United States (U.S.) in 2009, with WNS documented in Tennessee the winter of 2011 [30].

Foraging during intermittent arousals from hibernation may benefit bats at winter roost sites infected with *Pd* by augmenting energy reserves. In the southeastern U.S., bats remain active throughout winter regardless of *Pd* prevalence, with levels of activity corresponding to ambient temperature at dusk [31, 32]. As *Pd* became established in the southeastern U.S., daytime and cold temperature flights became more prevalent [31], yet less than 50% of the bats captured at these sites were positive for *Pd* and WNS, suggesting that winter activity was not driven by infection [33]. Although some active bats likely arouse throughout winter because of the physiological effects of WNS, many individuals with mild or no infection may be taking advantage of warm winter nights and higher prey availability to increase their energy reserves during hibernation. Therefore, it is important to determine what WNS susceptible bats are consuming during winter to better inform conservation actions implemented to help minimize disease related declines.

To date, the winter diet of North American bat species has been identified using traditional morphological methods [13, 18–20]. However, molecular methods, such as Next Generation sequencing of DNA amplified from fecal pellets, allow for a more complete view of the diet of insectivorous bats [34]. Although molecular techniques have been used to elucidate the diet of several insectivorous bat species [34–37], these studies have all been conducted during warm seasons. Our study is the first to examine the diet of North American insectivorous bat species captured outside of caves during winter. The rapid rates of digestion and defecation among bats [38] led us to infer that the fecal pellets provided by bats captured outside of hibernacula in winter (October–April) would contain insect remains consumed during bouts of winter foraging. The objectives of our study were to (1) identify the dietary composition of bats captured throughout winter, (2) compare the composition of recently consumed prey among bat species, and (3) determine how the composition of each species’ diet varied throughout the hibernation period (i.e., early-, mid-, and late-hibernation).

## Materials and methods

### Study area

**Fig. 1:**
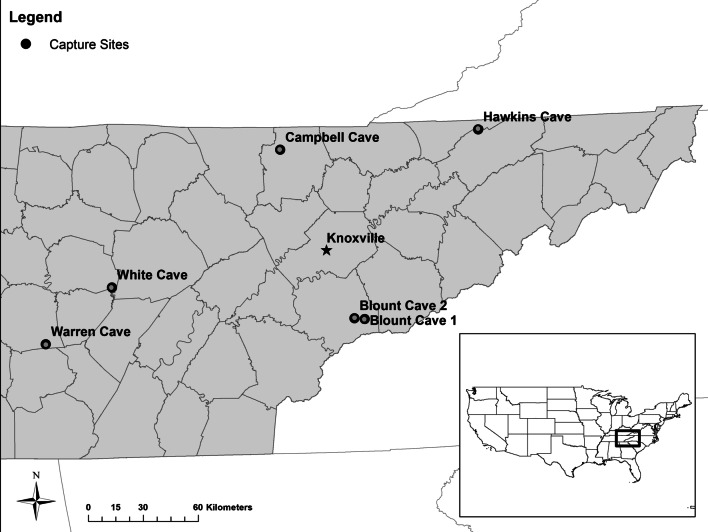
Bat capture sites at cave hibernacula in middle and east Tennessee, U.S. Bats were captured during hibernation (October–April) 2012/2013–2017/2018 at six cave sites using mist-nets deployed at least once per month for five hours starting 30 min before civil sunset

During five winters (i.e., October–April of 2012/2013, 2013/2014, 2015/2016, 2016/2017, and 2017/2018), we collected fecal samples from bats captured at six hibernacula in middle and eastern Tennessee (Fig. 1). Two hibernacula in central Tennessee (Warren and White Caves) were located on the Cumberland Plateau, at 340–350 m elevation. Two northeastern hibernacula (Campbell and Hawkins Caves) were located within the Cumberland Mountains, both at approximately 450 m elevation. Blount Cave 1 and 2 were located in the Great Smoky Mountains National Park (GRSM) at 525 m elevation. Mean nightly winter temperatures outside hibernacula, measured from 30 min before dusk to 30 min after dawn using HOBO U-series data loggers (Onset Computer Corporation, Bourne, MA, USA), ranged from − 2.03 to 20.16 °C.

### Bat captures

We deployed single-, double- and triple-high mist nets (Avinet Inc., Dryden, NY, U.S.; mesh diameter: 75/2, 2.6 m high, 4-shelves, 6–12 m wide) at hibernacula entrances or across forest corridors within 100 m of hibernacula at least once per month from October – April during each winter of the study. We opened mist nets 30 min before civil sunset and left them open for five hours, or until we captured 30 bats or ambient temperature dropped below 0 °C. After capture, we placed bats in individual brown paper bags and held them for 30–60 min in an insulated box with four hand-warmers (HotHands®, Dalton, GA, U.S.) to provide time for defecation. After holding, we identified each captured individual to species and determined sex, reproductive condition, forearm length (mm), weight (g), and wing-damage index (WDI, Reichard and Kunz 2009). We collected fecal pellets from the paper bags using sterile tweezers. We stored fecal pellets in 2.0 ml microcentrifuge tubes (Fisherbrand®, Fisher Scientific Co. L.L.C, Pittsburgh, PA, U.S.) with indicating silica gel desiccant (Grade 48, 4–10 mesh; Fisherbrand®, Fisher Scientific Co. L.L.C, Pittsburgh, PA, U.S.; [39]). Samples were frozen at -20 °C the night of collection and stored until analysis.

Field research followed U.S. Fish and Wildlife Services (USFWS) WNS Decontamination Guidelines [40]. All capture and handling techniques were approved by the University of Tennessee Institute of Animal Care and Use Committee (IACUC 2026–0514 and IACUC 2253–0320) and were consistent with the guidelines put forth by the American Society of Mammalogists (Sikes and ACUC 2016). We obtained federal (USFWS TE-71613A and USFWS TE-35313B, GRSM-2013-SCI1053; GRSM-2014-SCI1053, GRSM-2016-SCI1253, GRSM-2018-SCI1253) and state (TWRA 3716, TWRA 3741, TDEC 2009–038, TDEC 2011–031) permits to capture and handle bats and collect samples at winter hibernacula.

### Dietary analysis

The winter collection period was divided into three periods, which corresponded to the depth of winter (documented by mean nightly temperature) in Tennessee: early-hibernation = October – November (14.01 °C ± 1.11 SE); mid-hibernation = December–February (7.30 °C ± 1.05SE); and late-hibernation = March–April (14.52 °C ± 1.09 SE). Fecal samples used in this analysis were selected based on size of sample (2–4 pellets, up to 50 mg), species, sex, site, and winter collection period. We extracted DNA from feces using PowerSoil® (MO BIO Laboratories, Inc., Carlsbad, CA, U.S.) or QIAamp PowerFecal (Qiagen, Germantown, MD, U.S.) DNA Isolation Kits following the manufacturers’ protocols with one minor modification of increasing the first incubation period at 4 °C from 30 min to 12 h, to enhance removal of pigment from the DNA extraction product. We included one reagent-only control in each set of reactions (i.e., one control for every 24 samples) and stored extracted DNA at – 20 °C prior to Polymerase Chain Reaction (PCR) amplification. We targeted the mitochondrial cytochrome c oxidase 1 (CO1) gene using insect primers developed by Zeale et al. [41] (ZBJ-ArtF1c and ZBJ-ArtR2c). Conditions for PCR amplification and library prep were similar to those listed in Divoll et al. [42]. We amplified samples collected during the winters of 2012/2013 and 2013/2014 with primers modified for the Ion Torrent platform (Life Technologies, Carlsbad, CA, U.S.) with adapters and unique 10-base indexes, and samples collected during the winters of 2015/2016, 2016/2017, and 2017/2018 with primers containing adapters modified for the MiSeq platform (Illumina, San Diego, CA, U.S.).

During library preparation, we purified Ion Torrent samples with Agencourt AMPure XP beads (Beckman Coulter, Brea, CA, U.S.), prepared with an Ion Plus Fragment Library Kit (Life Technologies, Carlsbad, CA, U.S.), then size-selected for products of approximately 300 bp on the Pippin Prep (Sage Science, Beverly, MA, U.S.) before re-purifying with AMPure beads. We confirmed the quality and quantity of final products on a Bioanalzyer (Agilent Technologies, Santa Clara, CA, U.S.), pooled at approximately equimolar concentrations, loaded on an Ion 318 chip to run on the ION Torrent at the University of Tennessee Genomics Core. Library preparation for the Illumina MiSeq samples involved purifying the PCR products with Agencourt AMPure XP beads, dual-indexing with Illumina Nextera XT indexes, and re-purifying with Agencourt AMPure XP beads. We confirmed the quantity and quality of final products on a Bioanalzyer, pooled at approximately equimolar concentrations, and loaded at 6 pM with 20% PhiX on a v2, 500 cycle flow-cell reading 225 bases, paired-end, on the Illumina MiSeq at the University of Tennessee Genomics Core.

We analyzed sequences using the QIIME platform [43] and the workflows outlined in Divoll et al. [42] and Cravens et al. [44], with an additional step to eliminate potentially chimeric sequences. Briefly, we demultiplexed samples and pooled sequences in the forward and reverse direction into one fasta file with the same orientation. We then clipped primer sequences and removed any sequence that did not contain both forward and reverse primer sequences from further analysis. We clustered sequences into operational taxonomic units (OTUs) using the SWARM method with a resolution of two. We extracted representative sequences from each OTU cluster to create an OTU table based on abundance and used the ‘usearch’ command with denovo filtering in QIIME 2 to remove potentially chimeric sequences from the clustered OTUs. We removed sequences shorter than 147 bp or longer than 167 bp, representing ten bases on each side of the expected 157 bp. Due to potential index jumping, we conservatively removed any sequence that appeared in an individual bat less than 10 times for Ion Torrent data and less than 50 times for MiSeq data.

We ran the representative set of sequences through the COI database in the Barcode of Life Database (BOLD; [45]) using the package “bold” [46] in R [47]. We considered the first 40 records for each representative OTU and removed records with ≤ 99% similarity and country of collection outside of the U.S. and Canada [42, 44]. Where more than one identification for an OTU was present at ≥ 99%, we deferred to the next highest level of taxonomy for identification (i.e., multiple species within an order, we deferred to the order). We collapsed unique OTUs assigned to the same taxonomy into a single OTU, representing one bat prey item. We also merged duplicate OTUs, identified via the same unique BOLD identification number or within 1–4 bp via Sequencher (Gene Codes Corporation, Ann Arbor, MI, U.S.). We ran sequences produced from both the IonTorrent and MiSeq through the BOLD identification pipeline together to ensure consistent taxonomic identifications.

### Statistical analysis

We calculated the relative read abundance (RRA) of OTUs (i.e., the proportion of reads per each prey item in each sample) for each bat species following descriptions by Deagle et al. [48] and Vesterinen et al. [49]. We assessed sample coverage using the iNEXT package [50] to determine accuracy in describing the diversity of arthropod prey in the bat diet with respect to our sampling effort [51–53]. We plotted extrapolation curves using the entire sample set with all OTUs, including those that were not identified to any taxonomic level in BOLD, and used species richness (Hill number; q = 0) as the diversity measure [50, 52]. We used the VEGAN package [54] to test for variation in prey composition among bat species and hibernation periods using ANOSIM (Analysis of Similarity; [55] and PERMANOVA (Permutational Multivariate Analysis of Variance, *adonis* function) tests with 999 permutations. We grouped prey by family or order to minimize zero-inflated counts. To visually inspect the variation in prey consumption by arthropod species, family, and order consumed across bat species and hibernation periods, we used the ordination technique of nonmetric multidimensional scaling (NMDS) in VEGAN. We used a Bray–Curtis dissimilarity distance measure to calculate distances among samples and assessed the fit of the NMDS by observing the “stress” value [54]. Finally, we used the bipartite package [56] to draw an interaction web for each bat species using RRA. All statistical analyses and visualizations were performed using R [47].

## Results

### Bat captures

We captured 2044 individuals of 10 bat species over five winters. None of the 13 *Lasionycteris noctivagans* (silver-haired bat) captured provided fecal samples, therefore our analysis includes feces from nine species. We collected 518 fecal samples, on 84 of the 154 successful capture nights, with 25% of bats captured providing feces (Table 1). The mean nightly temperature on nights that samples were provided was 9.85 °C ± 0.87 SE, with temperatures ranging from − 2.03 to 20.16 °C. Of the fecal pellets collected, we selected 284 fecal pellet samples (117 samples from 2012/2013 and 2013/2014, and 168 samples from 2015/2016, 2016/2017 and 2017/2018) that met our criteria for analysis (i.e., early-hibernation: n = 136, mid-hibernation: n = 38, late-hibernation: n = 110).

**Table 1:** Total number of bats captured in Tennessee during hibernation (October–April) 2012/2013 to 2017/2018

Species^a^	Total bats captured	Total fecal samples	% Fecal provided^b^	Samples analyzed^c^
*Corynorhinus rafinesquii* (CORA)	21	10	47.6	7
*Eptesicus fuscus* (EPFU)	121	18	14.9	11
*Lasiurus borealis* (LABO)	15	5	33.3	5
*Lasionycteris noctivagans* (LANO)	13	0	0	–
*Myotis grisescens* (MYGR)	754	156	20.7	65
*Myotis leibii* (MYLE)	288	130	45.1	85
*Myotis lucifugus* (MYLU)	54	17	31.5	9
*Myotis septentrionalis* (MYSE)	224	55	24.6	26
*Myotis sodalis* (MYSO)	350	110	31.4	65
*Perimyotis subflavus* (PESU)	204	17	8.3	10
Grand total	2044	518	25.3	283

Each row represents the total number of individuals captured per species, including the total number of fecal samples collected and analyzed

^a^CORA = Rafinesque’s big-eared bat, EPFU = big brown bat, LABO = red bat, LANO = silver-haired bat, MYGR = gray bat; MYLE = eastern small-footed bat, MYLU = little brown bat, MYSE = northern long-eared bat, MYSO = Indiana bat, PESU = tri-colored bat

^b^% Fecal provided: percent of individuals that provided a fecal sample

^c^Samples analyzed: total number of fecal pellets amplified and sequenced

### Dietary analysis

**Fig. 2:**
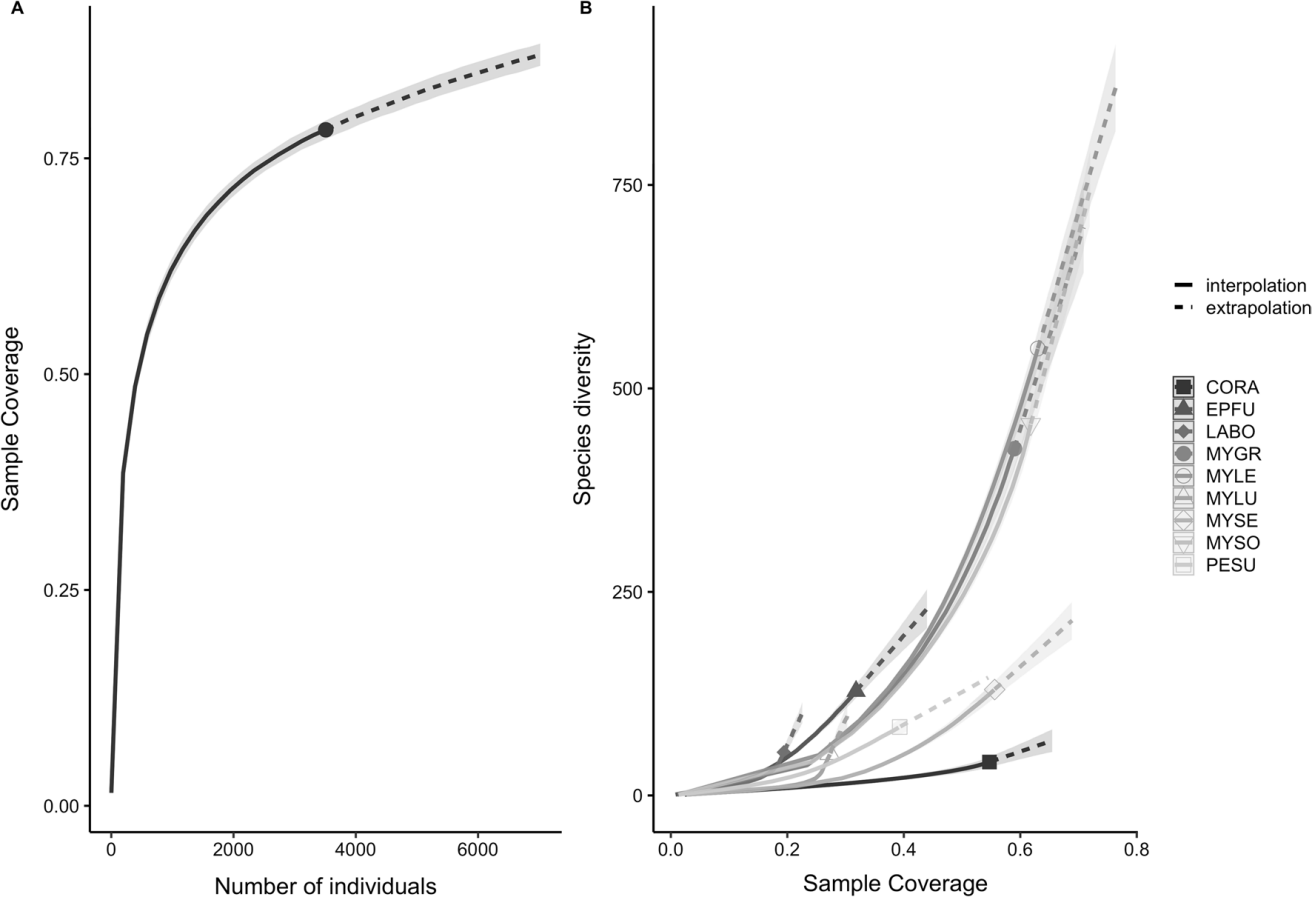
**a** Sample coverage for arthropod prey detected in all nine bat species captured throughout hibernation (October–April) 2012/2013–2017/2018 at cave sites in middle and east Tennessee, U.S. **b** Sample coverage by species diversity for each of the nine bat species captured. Interpolation (solid line segments) and extrapolation (dashed line segments) estimates of sample coverage were calculated as a function of sampling units produced in package iNEXT. We used Hill number q = 0 to calculate curves for species richness. 95% confidence intervals for each estimate are represented in shaded areas. Species acronyms: CORA: *Corynorhinus rafinesquii* (Rafinesque’s big-eared bat), EPFU: *Eptesicus fuscus* (big brown bat), LABO: *Lasiurus borealis* (red bat), MYGR: *Myotis griscesens* (gray bat), MYLE: *Myotis lebii* (eastern small-footed bat), MYLU: *Myotis lucifugus* (little brown bat), MYSE: *Myotis septentrionalis* (northern long-eared bat), MYSO: *Myotis sodalis* (Indiana bat)*,* and PESU: *Perimyotis subflavus* (tri-colored bat)

After bioinformatics processing and clean-up, we clustered and filtered sequencing reads to a total of 1209 unique OTUs. Of the unique OTUs, we identified 716 (59.2%) with matching sequences in BOLD, belonging to 14 orders, 134 families, and 587 genera or species (Additional file 2: Table S1). We were unable to identify the remaining 493 OTUs, likely due to a lack of reference sequences in BOLD and GenBank (Additional file 3: Table S2). Sample coverage of dietary diversity for all bat species amounted to 0.7832 (± 0.011 SE; Fig. 2a) and species diversity (Hill number q = 0) accumulation curve of arthropods detected in the diet in relation to the number of fecal samples was near its asymptotic point. Sample coverage for individual bat species varied (Fig. 2b). Of the unique OTUs, 762 were represented in (i.e., consumed by) one bat each, with the remaining 447 OTUs represented in two to 62 bats (Additional file 4: Table S3). The number of OTUs per bat fecal sample ranged from one to 58 with a mean of 10.44 ± 0.495 SE OTUs per fecal sample.

**Fig. 3:**
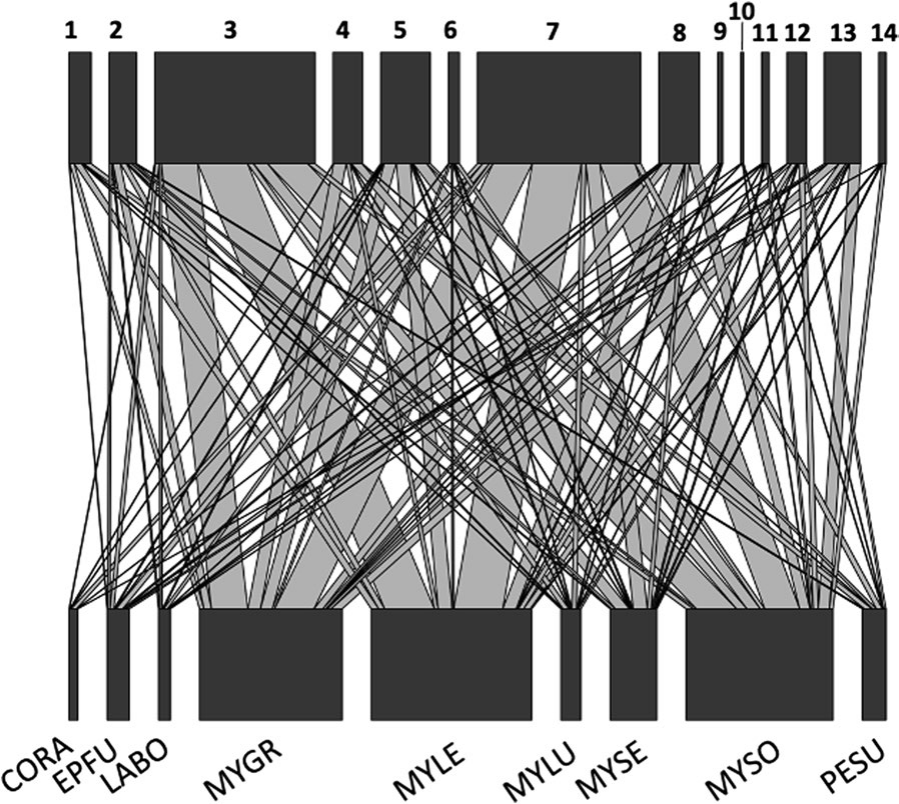
Food web of bat species and their arthropod prey species visualizing the similarities in the diet. The upper row represents arthropod orders consumed, with the blocks in the lower row the bat species. Lines connecting a bat species with an arthropod order represents the identification of consumption, and the thickness of the line represents the total number of times the order was represented in each bat species. Numbers correspond to 1: Araneae, 2: Coleoptera, 3: Diptera, 4: Ephemeroptera, 5: Hemiptera, 6: Hymenoptera, 7: Lepidoptera, 8: Neuroptera, 9: Orthoptera, 10: Plecoptera, 11: Psocodea, 12: Psocoptera, 13: Trichoptera, and 14: Trobidiformes. Unidentified OTUs were not included in this figure

The dispersion of diets varied weakly among bat species (PERMANOVA: R^2^ = 0.046, *P* = 0.001); however, diet composition was not significantly different (ANOSIM: R = 0.058, *P* = 0.400; Fig. 3). Diptera, Hemiptera, and Lepidoptera were consumed by every species of bat for which fecal samples were collected (n = 9). Over half of the samples of *Corynorhinus rafinesquii* (Rafinesque’s big-eared bat; 72.2%) and *Lasiurus borealis* (red bat; 67.4%) contained Lepidoptera. Diptera were the most common order of prey present in the diet of *M. leibii* (eastern small-footed bat; 32.4%), *M. lucifugus* (little brown bat; 37.4%), *M. sodalis* (Indiana bat; 35.5%) and *Perimyotis subflavus* (tri-colored bat; 68.8%). Coleoptera and Neuroptera were consumed by eight bat species; Ephemeroptera, Psocodea, Psocoptera and Trichoptera by seven bat species; Araneae and Hymenoptera by six bat species; Plecoptera and Trombidiformes by four bat species; and Orthoptera by three bat species (Table 2). Every species of bat consumed at least one OTU that was unidentified, comprising 7–33.4% of all reads.

**Table 2:** Top prey items consumed by nine bat species captured at caves in Tennessee throughout hibernation (October–April) 2012/2013 to 2017/2018

Prey OTUs			Bat species^a^									
Order	Family	Species	CORA	EPFU	LABO	MYGR	MYLE	MYLU	MYSE	MYSO	PESU	All species (ranked)
Diptera	Chironomidae	*Chironominae*				8						
Diptera	Drosophilidae	*Drosophila tripunctata*								8		
Diptera	Limoniidae	*Dicranomyia*				9	12			18		**1**
Diptera	Limoniidae	*Limoniidae*	1			8						
Diptera	Limoniidae	*Ormosia rubella*									3	
Diptera	Mycetophilidae	*Sciophila*									3	
Diptera	Psychodidae	*Psychoda*					10					
Diptera-309								2				
Diptera-341						16	18			22	3	**2**
Ephemeroptera	Ephemeridae	*Hexagenia limbata*								7		
Hemiptera	Aphididae	*Grylloprociphilus imbricator*								8		
Hemiptera	Derbidae	*Omolicna uhleri*								7		
Lepidoptera	Acrolepiidae	*Acrolepiopsis incertella*		3								
Lepidoptera	Blastobasidae	*Pigritia laticapitella*		3								
Lepidoptera	Coleophoridae	*Coleophora duplicis group*				8						
Lepidoptera	Cosmopteigidae	*Perimede erransella*				7						
Lepidoptera	Crambidae	*Palpita aenescentalis*	2									
Lepidoptera	Depressariidae	*Agonopterix pulvipennella*	3									
Lepidoptera	Depressariidae	*Machimia tentoriferella*					10			12		**8**
Lepidoptera	Erebidae	*Phoberia atomaris*	2						3			
Lepidoptera	Erebidae	*Sigela brauneata*		2								
Lepidoptera	Gelechiidae	*Chionodes thoraceochrella*			2	11	12	2	5	13		**6**
Lepidoptera	Gelechiidae	*Coleotechnites quercivorella*		2								
Lepidoptera	Gelechiidae	*Dichomeris*					9					
Lepidoptera	Gelechiidae	*Sinoe*		2								
Lepidoptera	Gelechiidae	*Sinoe chambersi*							6			**4**
Lepidoptera	Gelechiidae	*Sitotroga cerealella*									3	**10**
Lepidoptera	Geometridae	*Melanolophia canadaria*		2								
Lepidoptera	Nepticulidae	*Ectoedemia grandisella*		2								
Lepidoptera	Noctuidae	*Chrysodeixis includens*		2								
Lepidoptera	Noctuidae	*Eupsilia cirripalea*	3									
Lepidoptera	Noctuidae	*Lithophane grotei*	3									
Lepidoptera	Noctuidae	*Sericaglaea signata*	4									
Lepidoptera	Noctuidae	*Xestia elimata*	3									
Lepidoptera	Notodontidae	*Heterocampa guttivitta*							6			
Lepidoptera	Tortricidae	*Argyrotaenia velutinana*		4			9					
Lepidoptera	Tortricidae	*Chimoptesis gerulae*							3			
Lepidoptera	Tortricidae	*Decodes basiplagana*		2								
Lepidoptera	Tortricidae	*Pandemis limitata*					11					
Lepidoptera	Tortricidae	*Pseudexentera cressoniana*							7			
Lepidoptera	Tortricidae	*Pseudexentera sepia*	2						6			
Lepidoptera-609									3			
Neuroptera	Hemerobiidae	*Hemerobius*				7	14		4	7		
Unidentified-1074						7						**9**
Unidentified-1081						8						**7**
Unidentified-1092												**5**
Unidentified-1123							12		3			**3**
Unidentified-1179											4	
Unidentified-1230			3									

Numbers below each species acronym represent the total number of individuals found to have consumed the corresponding prey OTU. Prey OTUs that were not identified beyond Order or not identified, but were determined to be genetically distinct, are denoted with a unique ID number

^a^CORA = *Corynorhinus rafinesquii* (Rafinesque’s big-eared bat), EPFU = *Eptesicus fuscus* (big brown bat), LABO = *Lasiurus borealis* (red bat), LANO = *Lasionycteris noctivagans* (silver-haired bat), MYGR = *Myotis grisescens* (gray bat); MYLE = *Myotis leibii* (eastern small-footed bat), MYLU = *Myotis lucifugus* (little brown bat), MYSE = *Myotis septentrionalis* (northern long-eared bat), MYSO = *Myotis sodalis* (Indiana bat), PESU = *Perimyotis subflavus* (tri-colored bat)

**Fig. 4:**
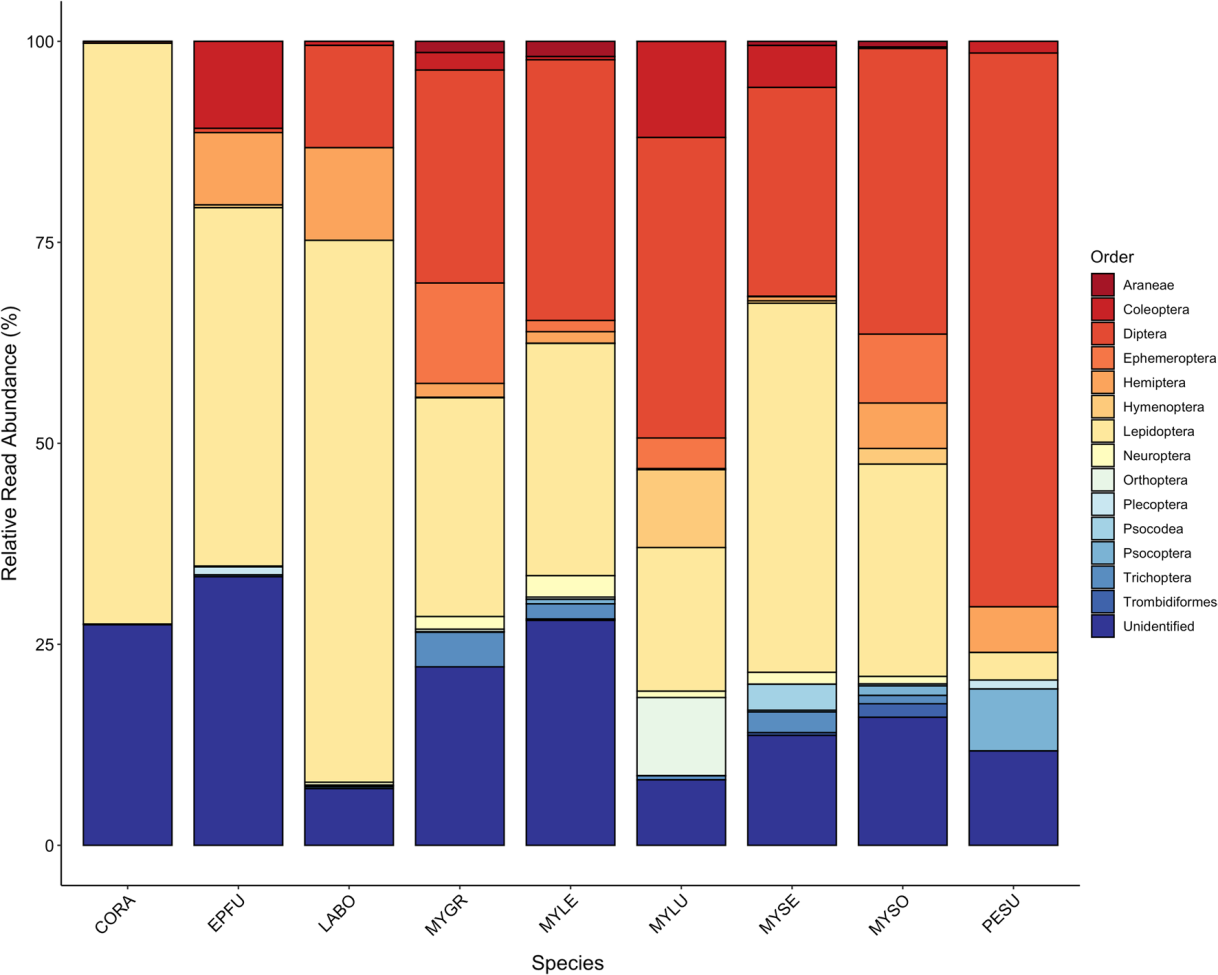
Relative read abundance (RRA) of arthropod Orders consumed by nine species of bat captured outside of caves in Tennessee during hibernation (October–April) 2012/2013 to 2017/2018. OTUs were identified to the finest taxonomic resolution possible using BOLD and GenBank. Those that were not identified are listed as “unidentified”. All years and seasons are combined. Species acronyms: CORA: *Corynorhinus rafinesquii* (Rafinesque’s big eared bat), EPFU: *Eptesicus fuscus* (big brown bat), LABO: *Lasiurus borealis* (red bat), MYGR: *Myotis griscesens* (gray bat)*,* MYLE: *Myotis lebii* (eastern small-footed bat), MYLU: *Myotis lucifugus* (little brown bat), MYSE: *Myotis septentrionalis* (northern long-eared bat*)*, MYSO: *Myotis sodalist* (Indiana bat), and PESU: *Perimyotis subflavus* (tri-colored bat)

*Myotis* species had the most diverse diets among the five genera of bats examined, ranging from 11 to 14 orders (Fig. 4). Excluding unidentifiable OTUs, *C. rafinesquii* had the least diverse diet (n = 6 orders), followed by *L. borealis* and *P. subflavus* (n = 8 orders), and *E. fuscus* (n = 11 orders). *Eptesicus fuscus* consumed the highest proportion of unidentifiable OTUs (33.4%).

The two Diptera species (*Dicranomyia*; Limoniidae) and an unknown Diptera were the most consumed OTUs detected in samples (Table 2). Four lepidopteran species were among the top 10 most common OTUs detected in samples, specifically, *Machimia tentoriferella* (Depressariidae), *Chionodes thoraceochrella* (Gelechiidae), *Sinoe chambersi* (Gelechiidae), and *Sitotroga cerealella* (Gelechiidae). Four additional OTUs, which were not identified to Order, were also represented in the top 10 prey OTUs detected.

Eight of the top 10 prey species detected in the diet of *C. rafinesquii* were lepidopterans with four representing the Family Noctuidae (Table 2). The most common OTUs for *E. fuscus* were all lepidopteran species, including *Argyrotaenia velutiana* (Tortricidae). At least 2 individuals from each species of *Myotis* consumed the lepidopteran, *Chionodes thoraceochrella* (Gelechiidae). The commonly detected OTU for *P. subflavus* was an unidentified OTU (Table 2).

**Fig. 5:**
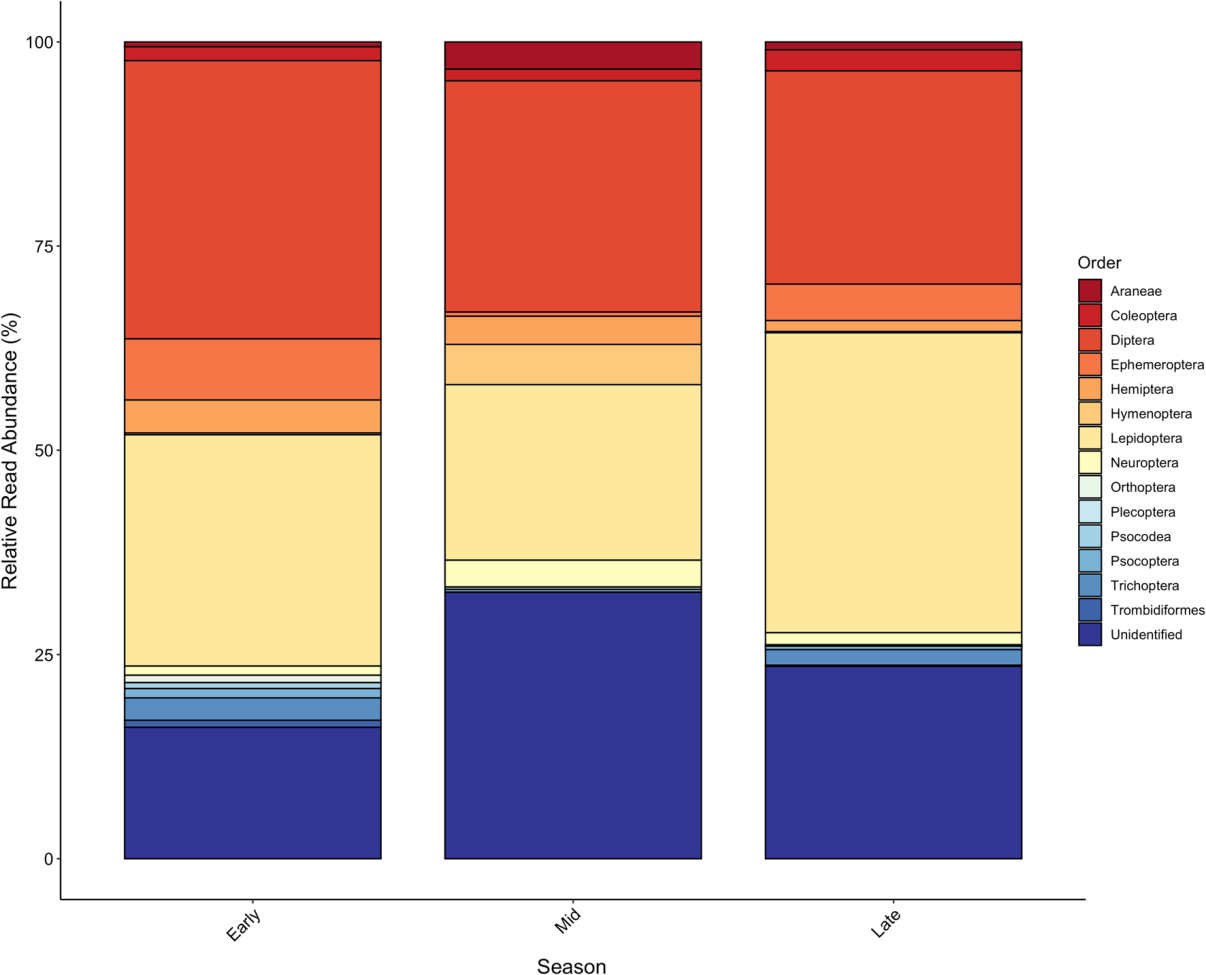
Relative read abundance (RRA) of arthropod Orders consumed by bats throughout three hibernation periods: early (October–November), mid (December–February) and late (March–April) of 2012/2013 to 2017/2018. OTUs were identified to the finest taxonomic resolution possible using BOLD and GenBank. Those that were not identified are listed as “unidentified”. All years and bat species are combined

The dispersion of diets varied among hibernation periods (PERMANOVA: R^2^ = 0.01246, *P* = 0.001); however, diet composition was not significantly different (ANOSIM: R = 0.049, *P* = 0.7; Fig. 5). All 14 orders were consumed throughout early- and late-hibernation. Twelve orders were consumed during mid-hibernation (December – February), where the most dominant orders were Diptera (28.3% of all reads) and Lepidoptera (21.5% of all reads). Dipteran and lepidopteran species were consistently the most common food source throughout hibernation, ranging from 26.1% (late hibernation) to 34.1% (early hibernation) for Diptera, and 28.3% (early hibernation) to 36.7% (late hibernation) for Lepidoptera. The largest proportion of unidentified sequenced reads were from mid-hibernation (31.6%). All orders except Orthoptera and Trombidiformes were consumed throughout winter.

## Discussion

We found that bats actively forage and consume a wide diversity of prey during winter. Augmenting energy stores may be particularly critical for hibernating bats due to the ability to supplement energy stores lost during hibernation. This may be even more important due to the additional energetic burden of *Pd* infection and manifestation of WNS*.* The similarity in the dietary breadth of insects consumed per species suggests that bats are not preferentially selecting large, calorie-rich prey [57, 58].

While opportunities for foraging during winter are likely a limiting factor when compared to insect abundance in summer, we found that the RRA of the two most common orders, Diptera and Lepidoptera, remained consistent across our sampling period (early to late hibernation). We also note that the presence of 14 insect orders and over 700 insect OTUs in the winter diet of bats is equivalent, if not greater, than what has been documented in molecular studies conducted in summer [34–36]. This provides another line of evidence that bats may be eating whatever is available to them during winter.

Although the dietary composition across bat species was not statistically different, the species-specific ‘preference’ of prey based on their respective ecologies were consistent with diet studies conducted in summer (see 34–36). *Corynorhinus rafinesquii* consumed the narrowest range of prey, seeming to focus primarily on lepidopteran species. This species of bat is a known moth specialist, gleaning individuals off of the surface of vegetation and other substrates, and has been found to predominately consume species in the families Noctuidae and Geometridae [59–63]. Although Noctuidae species comprised nearly half of the lepidopteran species detected in winter (n = 8/18 species represented), we also detected multiple species from the families Depressariidae, Erebidae, Geometridae and Tortricidae in samples from *C. rafinesquii*. We found three prey Orders, including Ephemeroptera, Hemiptera, and Trombidiformes, which, to our knowledge, have not been documented in previous dietary studies of the species (Additional file 4: Table S3). All representative species were consumed during early or mid-hibernation.

The remaining eight bat species we sampled are considered predominately generalist insect consumers [64], and we found that *Myotis* species consumed the most diverse prey items. A large proportion of the OTUs detected in samples from *M. grisescens* and *M. sodalis* were comprised of aquatic species, including Diptera, Ephemeroptera, Hemiptera, Neuroptera, Plecoptera and Trichoptera, including 168 and 167 representatives in the diet of the two species, respectively. While our study was conducted during winter, these findings are consistent with the foraging ecology of the two bat species [65, 66]. Visual identification of *M. sodalis* guano collected during the summer suggests the species frequently consumes Diptera, Coleoptera, Lepidoptera and Trichoptera [67–70]. Research on the summer diet of *M. grisescens* indicates that the species forages low over streams and other bodies of water [71]. As these streams do not typically freeze during winter in Tennessee, the preferred prey of *M. grisescens*, aquatic-based insects, appear to be available year-round in the Southeast [71]. Thus, the year-round availability of aquatic-based insects in the Southeast appears to enable the species to maintain opportunistic selection of their preferred prey base throughout hibernation [71].

Interestingly, Araneae species were most commonly consumed by *Myotis* species, ranging from an individual record in *M. lucifugus* (*Philodromus rufus vibrans*) to 16 Araneae species in *M. sodalis*. *Anyphaena pectorosa*, a species of ghost spider, was consumed by five *M. sodalis* during early hibernation. Interestingly, this species is typically active above-ground for a short period of time between mid-June to mid-July [72] and can often be seen in low foliage or under rocks [73]. Thus, the consumption of this and other Araneae species most likely occurs opportunistically while bats are roosting in hibernacula [74].

*Eptesicus fuscus*, the largest of the nine species captured, was once considered a coleopteran specialist [75, 76]. However, recent studies [35], including our own, place them more as generalist consumers throughout the year. Only five coleopteran species were consumed by *E. fuscus*, substantially less than the number of representatives consumed by *M. grisescens, M. leibii, M. septentrionalis,* and *M. sodalis.* The lack of coleopteran species in the diet of *E. fuscus* could be attributed to seasonal variation and availability [35]. Alternatively, the seemingly low proportion of Coleoptera in the diet, especially when compared to the proportion of unidentified OTUs consumed by *E. fuscus,* could be due to the specific primers we used or a lack of genetic information in BOLD for Coleopteran species active in winter.

The winter consumption of insects by bats has illuminated the need for further research on the year-round foraging behavior of this diverse taxonomic group, as well as the activity and abundance of insects active throughout winter. Although over 50% of the identified reads were dipteran and lepidopteran species, information on the specific behaviors of these insect orders during winter is lacking. We collected a substantial number of samples for both early and late hibernation (136 and 109 bats, respectively), indicating that favorable weather conditions and presumably increased prey availability allow for successful foraging. Although there is evidence that some lepidopteran species in temperate regions remain active as caterpillars [77–79], it is unclear how many species of adult lepidoptera remain active in winter. Both morphological and molecular dietary studies have identified adult Lepidoptera in the diet of bats during winter, with many of those studies conducted in climates with more severe winters than in the southeastern portion of the U.S. [13, 22, 80–82]. Ultimately, there remains a significant knowledge gap about the winter activity of insects in the southeastern U.S.

As we consider the conservation implications of periodic activity and foraging throughout winter, we must also be cognizant of the plasticity in torpor of temperate North American bats [5, 83]. Prior to the emergence of WNS in North America, the predominant theory was that most bat species entered hibernation in October and remained in hibernacula until March–April [31]. We provide evidence that several bat species in the Southeast forage for aerial insects during winter. This knowledge is critical for natural resource managers, as it may allow for novel conservation actions that could enhance survival of overwintering bats (e.g., insect habitat management or artificial lights near hibernacula to attract insect prey). By enhancing insect habitat and abundance near bat hibernacula, the energetic costs of foraging could be minimized, thereby increasing fat storage and survival, particularly amongst WNS-susceptible species. As we confront a rapidly warming climate, we expect winter activity of bats will increase in more northern latitudes [84, 85]. Therefore, increasing knowledge of the winter activity of bats will have importance even after WNS is deemed endemic in North America.

## Supplementary Information

**Additional file 1**

**Additional file 2: Table S1**. Of the unique OTUs represented in the diet of bats, we identified 587 OTUS to genera or species. Rows labeled in bold with a sample size represent OTUs that were only identified to Order. We were unable to identify 493 OTUs. Total sequence reads represents the number of times the OTU sequence was represented and can be used as a relative abundance with larger number indicating more DNA was represented. Represented signifies the number of bats found to have consumed each OTU.

**Additional file 3: Table S2**. FASTA file with all sequence reads, prior to the removal of duplicate or redundant IDs. This fasta file is the merged sequence file from the Ion Torrent and MiSeq samples.

**Additional file 4: Table S3**. This table highlights the relative read abundance of all OTUs consumed across all bat samples. Each bat sample is an individual identified by hibernation period (hiber), species and sex.

## Data Availability

All data are available as supplemental files.
