# Supplementary material for: Feasting, not fasting: winter diets of cave hibernating bats in the United States
Source: Front Zool. 2021 Sep 23;18:48. doi: 10.1186/s12983-021-00434-9 (PMC8461964; doi:10.1186/s12983-021-00434-9)
Supplement: Supplementary file 1 — Additional file 1 [file 12983_2021_434_MOESM1_ESM.docx]

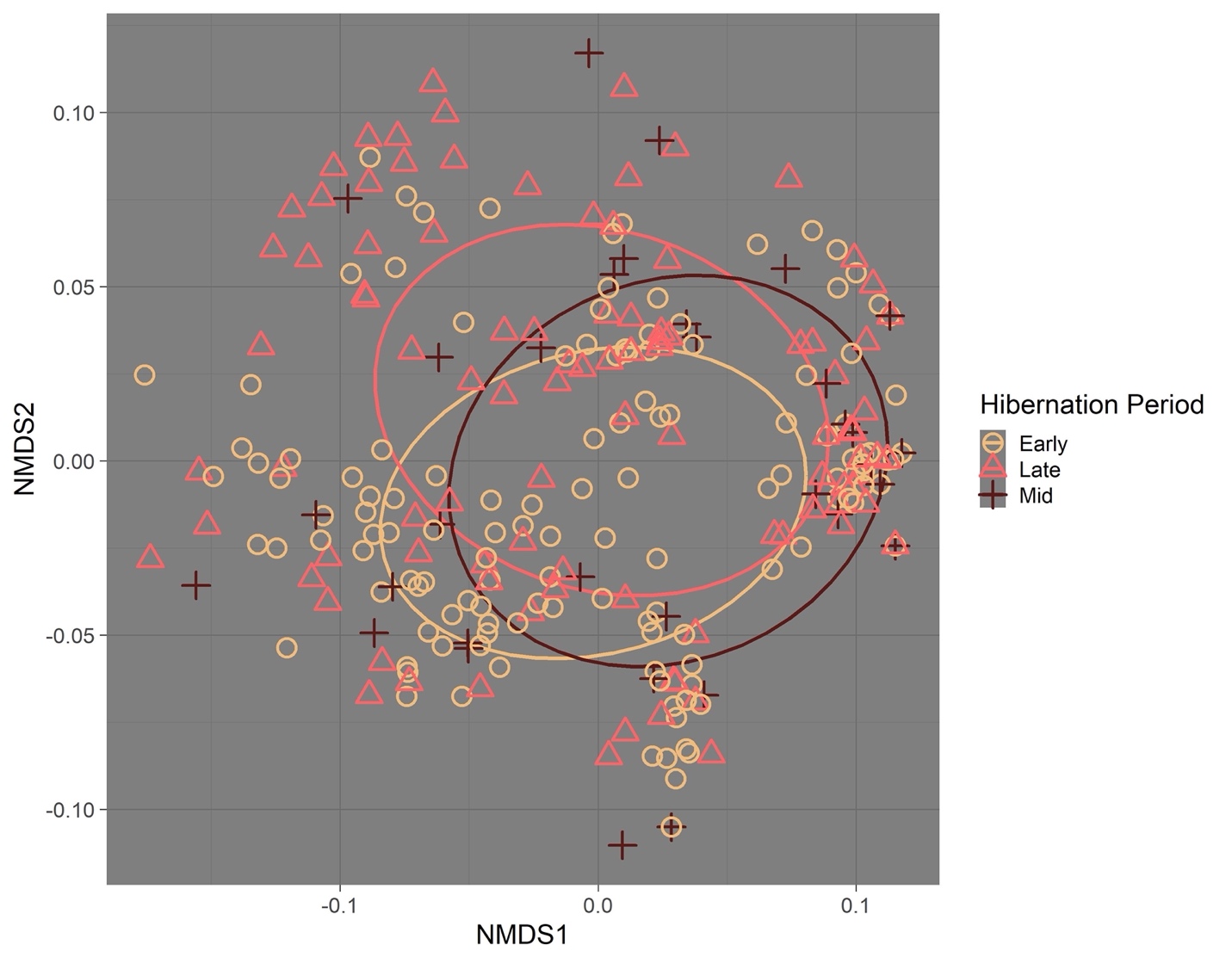
Supplemental Figure captions and table legends:

Supplemental Figure 1. NMDS plot representing the insect families (n = 134) consumed by all bat species (n = 9) for each hibernation period (early, mid and late) from 2012 – 2018. We found no significant difference in insect families consumed throughout hibernation as all families were consistently consumed in each period.


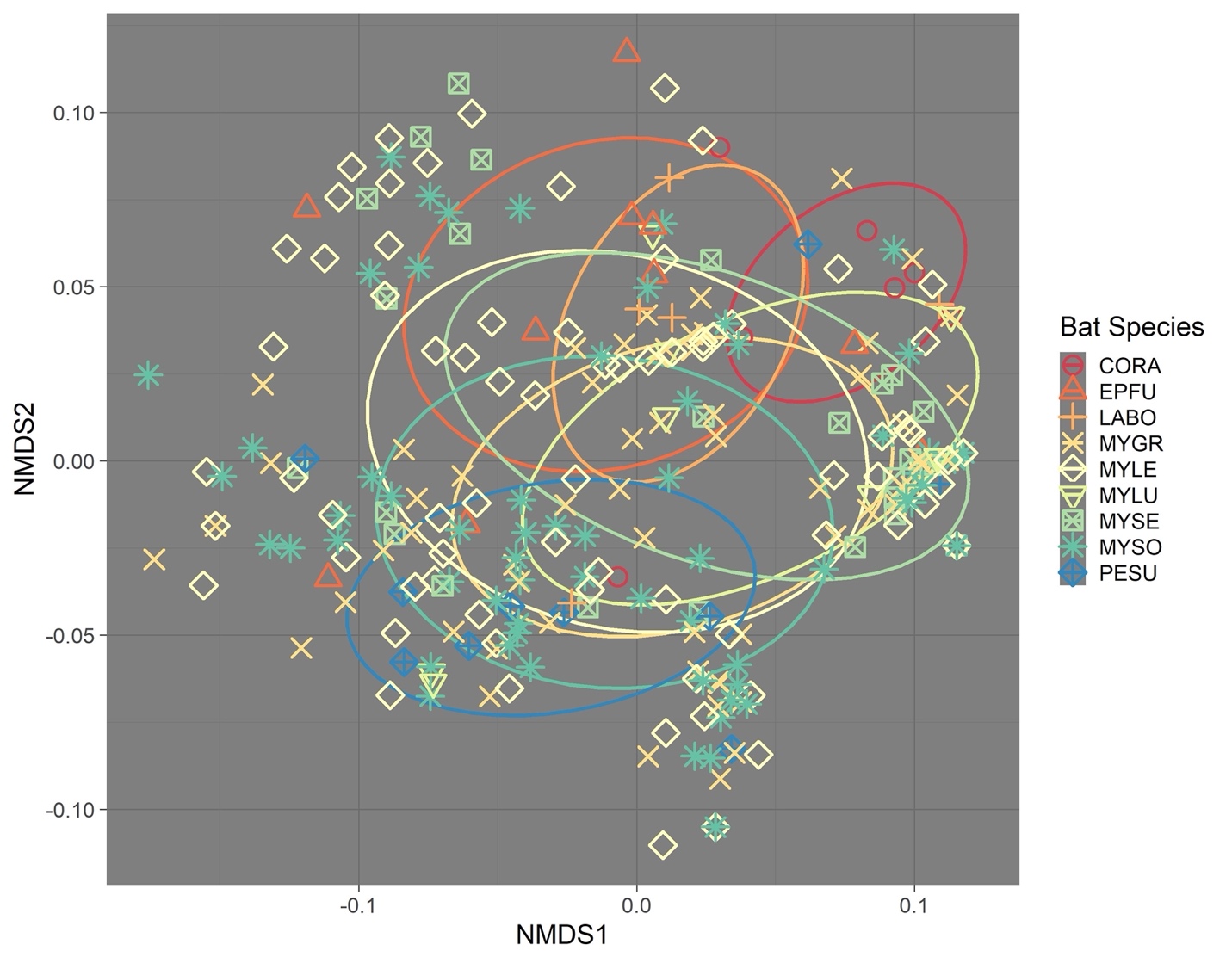
Supplemental Figure 2. NMDS plot representing the insect families (n = 134) consumed by all bat species (n = 9) across all sample years (winters 2012 – 2018). While we did find the breadth of insects consumed per bat species varied (i.e., CORA and LABO consumed fewer insect families than *Myotis* species), there was no significant difference in the composition of insect families consumed across bat species.
